# Functional studies of *McSTE24*, *McCYP305a1*, and *McJHEH*, three essential genes act in cantharidin biosynthesis in the blister beetle (Coleoptera: Meloidae)

**DOI:** 10.1093/jisesa/ieae070

**Published:** 2024-07-11

**Authors:** Yi Huang, Ling Shen, Fang Du, Zhongkang Wang, Youping Yin

**Affiliations:** Key Laboratory of Genetic Function and Regulation, School of Life Science, Chongqing University, Chongqing 400030, China; Key Laboratory of Genetic Function and Regulation, School of Life Science, Chongqing University, Chongqing 400030, China; School of Life Science, Jiangsu Normal University, Xuzhou 221116, China; Key Laboratory of Genetic Function and Regulation, School of Life Science, Chongqing University, Chongqing 400030, China; Key Laboratory of Genetic Function and Regulation, School of Life Science, Chongqing University, Chongqing 400030, China; Key Laboratory of Genetic Function and Regulation, School of Life Science, Chongqing University, Chongqing 400030, China

**Keywords:** *STE24*, *CYP305a1*, *JHEH*, gene function, anticancer

## Abstract

Cantharidin is a toxic defensive substance secreted by most blister beetles when attacked. It has been used to treat many complex diseases since ancient times and has recently regained popularity as an anticancer agent. However, the detailed mechanism of the cantharidin biosynthesis has not been completely addressed. In this study, we cloned *McSTE24* (encoding STE24 endopeptidase) from terpenoid backbone pathway, *McCYP305a1* (encoding cytochrome P450, family 305) and *McJHEH* [encoding subfamily A, polypeptide 1 and juvenile hormone (JH) epoxide hydrolase] associated to JH synthesis/degradation in the blister beetle *Mylabris cichorii* (Linnaeus, 1758, Coleoptera: Meloidae). Expression pattern analyses across developmental stages in adult males revealed that the expressions of 3 transcripts were closely linked to cantharidin titer exclusively during the peak period of cantharidin synthesis (20–25 days old). In contrast, at other stages, these genes may primarily regulate different biological processes. When RNA interference with double-stranded RNA suppressed the expressions of the 3 genes individually, significant reductions in cantharidin production were observed in males and also in females following *McJHEH* knockdown, indicating that these 3 genes might primarily contribute to cantharidin biosynthesis in males, but not in females, while females could self-synthesis a small amount of cantharidin. These findings support the previously hypothesized sexual dimorphism in cantharidin biosynthesis during the adult phase. *McCYP305a1* collaborates with its upstream gene *McSTE24* in cantharidin biosynthesis, while *McJHEH* independently regulates cantharidin biosynthesis in males.

## Introduction

Blister beetles (Coleoptera: Meloidae) are traditionally used to treat miscellaneous diseases. The dried body of the Chinese blister beetle, *Mylabri*s, has been used medicinally for more than 2,000 years in China (Wang 1989). Cantharidin is a defensive toxin predominantly produced by blister beetles and smaller oedemerid beetles (Coleoptera: Oedemeridae) as well as the major medicinal component of blister beetles (Carrel et al. 1986b, 1993, Mccormick and Carrel 1987). Cantharidin and its derivatives have curative effects on liver, lung, esophageal, and stomach cancers (Yang et al. 2007, Liu and Chen 2009). *Mylabris cichorii* and *Mylabris phalerata* (Pallas, 1782, Coleoptera: Meloidae) 2 pharmaceutical Meloidae species recorded in Chinese Pharmacopeia (National Pharmacopoeia Commission of China 2005), have been widely utilized to extract cantharidin for medical use. Most blister beetles demonstrate sexual dimorphism in terms of cantharidin production in the adult phase; females do not synthesize cantharidin after the larval stage. During mating, male beetles synthesize and transfer large pockets of cantharidin to their partners. Females pass the cantharidin to their eggs to protect them from natural predators (Carrel et al. 1993, Eisner et al. 1996). In *M. cichorii*, male cantharidin levels are low at the early stage after emergence, increase during days 20–25, and peak at 30 days after emergence, while at the same stage, the cantharidin levels remain low in females (Supplementary Table S1) (Wang 2008).

Early studies showed that trans-farnesol and mevalonic acid are 2 specific precursors in the biosynthesis of cantharidin (Schlatter et al. 1968, Guenther et al. 1969, Peter et al. 1977a, 1977b, Wolf-Dietrich et al. 1983). Huang et al. (2016) previously found that cantharidin biosynthesis in *M. cichorii* only via the mevalonate (MVA) pathway, which has been also demonstrated in other Meloidae species (Lü et al. 2016, Du et al. 2021). 3-hydroxy-3-methyl glutaryl coenzyme A reductase (HMGR) and farnesyl pyrophosphate synthase (FPPS) are rate-limiting enzymes in the MVA pathway, and in sesquiterpenoid biosynthesis (Manzano et al. 2004, Szkopińska and Płochocka 2005, Suzuki et al. 2010), *HMGR* and *FPPS* genes were proved to be act in cantharidin biosynthesis (Lü et al. 2016, Zha et al. 2017). Huang et al. identified 12 genes from the MVA pathway and its related downstream pathways that potentially regulate cantharidin biosynthesis (Figure 6 and Table 2 in Huang et al. 2016). Among these genes, *STE24*, which encodes the STE24 endopeptidase (a downstream gene of *HMGR* and *FPPS*), is the only gene locates in a branch pathway from farnesyl diphosphate to trans-farnesol, suggesting its potential role in cantharidin biosynthesis (Huang et al. 2016). *STE24* exhibited high expression during the peak cantharidin synthesis stage in male *M. cichorii*, with expression levels being inhibited after knockdown of its upstream gene *FPPS* (Zha et al. 2017). STE24 was first identified in yeast, where it acts in the production of a α-mating pheromone (Tam et al. 1998) and was also demonstrated essential for prenylation-dependent post-translational modification in eukaryotic organisms (Zhang and Casey 1996). *STE24* transcripts was differentially expressed between second-instar (the developmental origin of sexual dimorphic metamorphosis) male and female larvae in *Ericerus pela* (Chavannes, 1844, Hemiptera: Coccidae) (Liu et al. 2019), indicating that *STE24* might also closely related to juvenile hormone (JH) synthesis. However, whether *STE24* is directly related to cantharidin biosynthesis in blister beetles has never been studied.

Early research also suggested that cantharidin may be a metabolite of JH (Mccormick et al. 1986) since both JH and cantharidin can be blocked by 6-fluoromevalonate (Carrel et al. 1986b, Mccormick and Carrel 1987), besides cantharidin and JH may share a portion of pathway beyond farnesol. Jiang et al. (2017a) demonstrated that *EcMFE* (methyl farnesoate epoxidase, a cytochrome P450 enzyme) in *Epicauta chinensis* (Laporte, 1840, Coleoptera: Meloidae) acts in cantharidin biosynthesis. Huang et al. identified one gene encode cytochrome P450, family 305, subfamily A, polypeptide 1 (CYP305a1) that epoxidize methyl farnesoate to generate JH terminal enzyme (JH epoxide hydrolase) for JH biosynthesis (Huang et al. 2016). Since enzymes from cytochrome P450 family are not conserved between insect/blister beetle species (Jiang et al. 2017a, Fratini et al. 2021), to characterize whether this CYP450 identified in *M. cichorii* relates to cantharidin biosynthesis is needed. JHEH is a key enzyme involved in the degradation of JH (Share and Roe, 1988). Recent studies have also shown that cantharidin biosynthesis is linked to genes involved in JH synthesis or degradation (Huang et al. 2016, Jiang et al. 2017a). Huang et al. identified 4 *JHEH* unigenes (partial sequences of different *JHEH* isoforms), including one that displayed sex-specific expression, hinting at its potential role in cantharidin biosynthesis.

The renewed interest in the medical potentials of cantharidin has led to a blossoming of studies devoted to revealing the molecular mechanism of *in vivo* cantharidin synthesis, identifying key pathways such as “Terpenoid backbone biosynthesis” and “insect hormone biosynthesis” related to cantharidin biosynthesis (Liao et al. 2015, Huang et al. 2016, Lü et al. 2016, Jiang et al. 2017a, Zha et al. 2017, Du et al. 2021, Fratini et al. 2021, Zhou et al. 2023), and revealing the potential organ—fat body as the origin of cantharidin synthesis processing (Jiang et al. 2017b). However, most previous functional studies solely focused on genes either in terpene compound pathways (Lü et al. 2016, Zha et al. 2017) or in JH related pathways, and mainly focusing on males (Jiang et al. 2017a, Fratini et al. 2021). No study addresses the following questions: (i) Are the genes from terpenoid backbone pathway and JH biosynthesis and/or degradation pathway jointly or independently regulating cantharidin biosynthesis? (ii) Whether females could also synthesis cantharidin or if there are any genes participating in it?

**Fig. 1.**
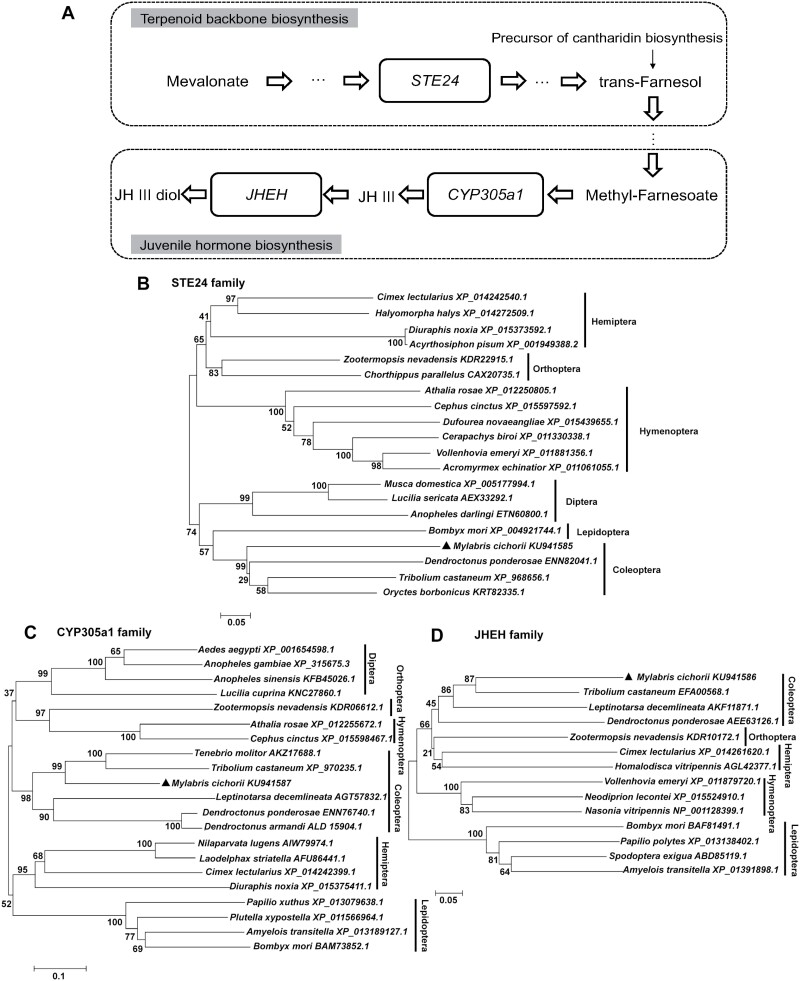
A) The relationship between *McSTE24*, *McCYP305a1*, and *McJHEH* in *M. cichorii. STE24* is located at a branch pathway from farnesyl diphosphate to trans-farnesol (the precursor of cantharidin biosynthesis) and is an upstream gene of *CYP305a1* and *JHEH*. *JHEH* is a downstream gene of both *STE24* and *CYP305a1*, locates at the juvenile hormone (JH) degradation pathway. *CYP305a1* epoxidizes methyl farnesoate to generate JH terminal enzyme (JH epoxide hydrolase) for JH biosynthesis. B–D) Phylogenetic analysis of insect STE24, CYP305a1, and JHEH proteins based on their corresponding amino acid sequences. Sequences are labeled with the species Latin names plus their GenBank accession numbers. Bootstrap values (1,000 replicates) are displayed by the notes. The genetic distance is drawn to scale.

In this study, we cloned 3 genes encoding STE24, CYP305a1, and JHEH. The relationship of 3 genes (*STE24*, *CYP305a1*, and *JHEH*) were summarized in Fig. 1A. *STE24* located at the terpenoid backbone biosynthesis pathway is an upstream gene of *CYP305a1* (associated with JH biosynthesis) and *JHEH* (related to JH degradation), 2 genes located at the juvenile hormone biosynthesis pathway, while *JHEH* is a downstream gene of *STE24* and *CYP305a1*. By RNAi knockdown to inhibit the expressions of 3 genes, we demonstrated that these genes are critical for cantharidin biosynthesis, primarily in males. We found that female adults could also synthesize a small amount of cantharidin. Our results suggested *CYP305a1* collaboratively regulates cantharidin synthesis with its upstream gene *STE24*. Conversely, *JHEH* appears to independently affect cantharidin biosynthesis, separate from the regulatory influence of either *CYP305a1* or *STE24* in *M. cichorii*.

## Materials and Methods

### Beetle Rearing

Wild *M. cichorii* were collected from Luodian, Guizhou Province, China, and then reared in our laboratory as previously described (Huang et al. 2016). Ethical review and approval were not required for the study on animals in accordance with the local legislation and institutional requirements. The adults used in the present study were first-generation beetles and were reared with sex segregation.

### Molecular Cloning of *McSTE24*, *McCYP305a1*, and *McJHEH*

Total RNA was extracted from the whole bodies of 20–25 days old adult males with TRIzol Reagent (Invitrogen, USA), treated with DNase I (Thermo Scientific, USA), and reverse transcribed to cDNA with a RevertAid First-Strand cDNA Synthesis Kit (Thermo Scientific, USA). The genomic DNA was extracted with the E.I.N.A. Insect DNA Kit (Omega, USA). 3ʹ- and 5ʹ-Genome walking was performed with a fusion primer and nested integrated PCR (FPNI-PCR) (Wang et al. 2011). A 3ʹ-Full RACE Core Set with PrimeScript RTase Kit (TaKaRa, Japan) was used to transcribe the first-strand cDNA for 3ʹ-random amplification of cDNA ends (3ʹ-RACE). To confirm the cDNA sequences assembled from the overlapping PCR products, the entire coding regions of the 3 genes were amplified from the cDNA library with the primers STE24-ORF-L/STE24-ORF-R (*STE24*), CYP305a1-ORF-L/CYP305a1-ORF-R (*CYP305a1*), and JHEH-ORF-L/JHEH -ORF-R (*JHEH*). All the PCR products were subcloned into the pMD19-T plasmid (TaKaRa, Japan). The cloning vector was amplified in *Escherichia coli* DH5α cells and then sequenced.

The PCR primers used in FPNI-PCR and 3ʹ-RACE were designed based on the unigenes of the 3 genes retrieved from the transcriptomic library of *M. cichorii* (Huang et al. 2016) (Supplementary Table S2). Universal primers are also shown in Supplementary Table S2.

### Sequence Analysis

Amino acid sequences of 3 genes were predicted by ORF Finder (http://www.ncbi.nlm.nih.gov/gorf/gorf.html) based on the cDNA sequences obtained by molecular cloning. Protein sequence analysis was performed using the ExPASy ProtParam (http://www.expasy.org/tools/ protparam.html), ExPASy ProtScale (http://web.expasy.org/protscale/), NPSA GOR4 (https://npsa-prabi.ibcp.fr/cgi-bin/npsa_automat.pl?page=npsa_gor4.html), SignalP (http://www.cbs.dtu.dk/services/SignalP/) and TMHMM (http://www.cbs.dtu.dk/services/TMHMM/). Sequence alignments were performed by DNAMAN v6. And the phylogenetic tree was compiled using MEGA 5.1 software.

### Stage-Specific Expression Pattern of *McSTE24*, *McCYP305a1*, and *McJHEH* in Male and Female Adults

Sex-segregated adults (*n* = 3) in different developmental phases after emergence (5-, 10-, 15-, 20-, 25-, and 30-day-old adult males and females) were used for a timed developmental gene expression analysis performed by Quantitative Reverse Transcription PCR (RT-qPCR).

RT-qPCR was performed on a CFX96 Real-Time PCR Detection System (Bio-Rad, USA) using GoTaq qPCR Master Mix (Promega, USA) to detect the gene expression changes after RNAi. The assays were run at 95 °C for 3 min, followed by 40 cycles at 95 °C for 10 s, 58 °C for 20 s, and 72 °C for 15 s. A reaction melting-curve analysis from 65 °C to 95 °C was then applied to all the reactions to ensure the consistency and specificity of the amplified products. Three biological replicates and triplicate reactions of each sample were conducted. The results were normalized to the expression levels of the internal reference genes *UBE3A* (ubiquitin-protein ligase E3A) and *RPL22e* (ribosomal protein) (Wang et al. 2014). All the primers used for the expression pattern analysis are listed in the Supplementary Table S3. The expression ratios were calculated from the cycle threshold values with the 2^−△△CT^ method (Livak and Schmittgen 2001).

### Double-stranded RNA-Based RNA Interference (RNAi) Knockdown and Determination of RNAi Efficacy

RNA interference (RNAi) was used to assess the roles of *McSTE24*, *McCYP305a1*, and *McJHEH* in the biosynthesis of cantharidin in *M. cichorii*. Double-stranded RNAs (dsRNAs) were designed based on full-length cDNA and synthesized with the MEGAscript RNAi Kit (Ambion, USA), according to the manufacturer’s instructions.

Three pairs of gene-specific primers with a T7 promoter region were designed to synthesize 504 bp, 598 bp, and 511 bp dsRNAs of *McSTE24*, *McCYP305a1*, and *McJHEH*, respectively. The enhanced green fluorescent protein gene (*eGFP*) and ddH_2_O were used as the negative control and blank control, respectively. Plasmid C with Bar-*eGFP* was used as the template to synthesize *eGFP*-dsRNA. The primers used for dsRNA synthesis are shown in Supplementary Table S4.

Because cantharidin biosynthesis peaks during 20–25 days in adult *M. cichorii* males, 19-day-old adult males and females were used in the RNAi experiment. The beetles were placed on ice and anesthetized for several minutes until they became inactive. dsRNA (15 μg in 5 μl) or equivalent ddH_2_O was injected into each body cavity from the second intersegmental membrane with a microinjector. Beetles were collected at 24 and 48 h after treatment (3 pools per sample, *n* = 3). The samples were immediately frozen in liquid nitrogen and stored at −80 °C for further use.

### Quantification of Cantharidin by GC-MS

RNAi was performed as described above. Beetles were collected from each group at 24 h after injection and dried in a 60 °C oven for 7 days to obtain a constant weight. Cantharidin was extracted from all the samples as previously described (pooled 15 beetles per sample, *n* = 3) (Ray et al. 1979, Capinera et al. 1985). The extracts were quantified with gas chromatography-mass spectrometry (GC-MS) on Agilent 7890A/5975C (USA), with an HP-5MS 5% phenyl methyl silox capillary column (30 m × 0.25 mm × 0.25 μm) and selected ion monitoring at *m*/*z* 76, 96, and 128. The amounts of cantharidin in the extracts were determined based on calibration curves that compared cantharidin standards in the range of 50–2,000 ng. The GC conditions were: initial temperature of 110 °C for 1 min, followed by a temperature increase of 3 °C/min up to 160 °C, then by 9 °C/min up to 220 °C, and then the temperature was maintained at 220 °C for 3 min. The detector (FID) temperature was set at 325 °C. Helium was used as the carrier gas. The total flow rate was 34 ml/min and the septum purge flow rate was 3 ml/min. The split ratio was 30:1. The MS conditions were: electron impact mass spectra were collected at an ionizing voltage of 70 eV, with the separator at 230 °C and the source at 150 °C; splitless injection (1 μl) was used. The abundance of standard cantharidin samples at 0.05, 0.1, 0.5, and 2 mg/ml were measured to calculate the standard curve based on the corrected area at the peak, cantharidin contents of each sample were then calculated and calibrated by the dried beetle body masses in mg/g.

### Detection of Expression Changes in Downstream Genes after Knockdown of *McSTE24* and *McCYP305a1*

RT-qPCR was employed to assess the alternations in the expression levels of downstream genes (*McCYP305a1* and *McJHEH*) following the knockdown of gene *McSTE24*, and similarly, the expression of the downstream gene (*McJHEH*) influenced by *McCYP305a1* knockdown.

### Statistical Analysis

All data were analyzed with one-way ANOVA (comparisons > 3 groups) followed by a post hoc Tukey’s test or *t*-test (comparison between 2 groups) using Graphpad Prism 9. Results were presented as mean ± SEM.

## Results

### Cloning and Sequence Analysis of *McSTE24*, *McCYP305a1*, and *McJHEH*

The sequences of *McSTE24* (GenBank accession number KU941585) and *McJHEH* (GenBank accession number KU941586) were obtained with a combination of 5ʹ-FPNI-PCR and 3ʹ-RACE strategies, and that of *McCYP305a1* (GenBank accession number KU941587) was obtained with 5ʹ- and 3ʹ-FPNI-PCR. The full-length sequences of *McSTE24, McCYP305a1*, and *McJHEH* are 1302, 1479, and 1380 bp, respectively, and encode proteins of 433, 492, and 459 amino acids, respectively (Supplementary Fig. S1). An alignment analysis of the McSTE24, McCYP305a1, and McJHEH proteins showed relatively high sequence identities to *Tribolium castaneum* (Herbst, 1797, Coleoptera: Tenebrionidae) *STE24* (XP_968656.1), *Tenebrio molitor* (Linnaeus, 1758, Coleoptera: Tenebrionidae) *CYP305a1* (AKZ17688.1), and *T. castaneum JHEH* (EFA00568.1) (59%, 62%, and 60%), respectively (Supplementary Fig. S2). The sequence alignment also demonstrated that McSTE24, McCYP305a1, and McJHEH are highly conserved relative to their corresponding homologs in other insects. The McSTE24 protein contains a HEXXH Zn^2+^-metalloprotease signature; McCYP305a1 contains the conserved motifs hinge, I helix, helix K, and heme binding loop; and McJHEH contains residues that potentially form the catalytic triad and oxyanion hole. Phylogenetic analysis revealed that McSTE24, McCYP305a1, and McJHEH belong to the STE24, CYP305a1, and JHEH families. They clustered together with other Coleoptera insects on the same or closely related branch (Fig. 1B–D). We therefore are confident that the cDNAs isolated in our study encode STE24, CYP305a1, and JHEH proteins in *M. cichorii*.

### *McSTE24*, *McCYP305a1*, and *McJHEH* Had Different Expression Patterns in Adult *M. cichorii* and Exhibited Time- and Sex-Specific Manners

**Fig. 2.**
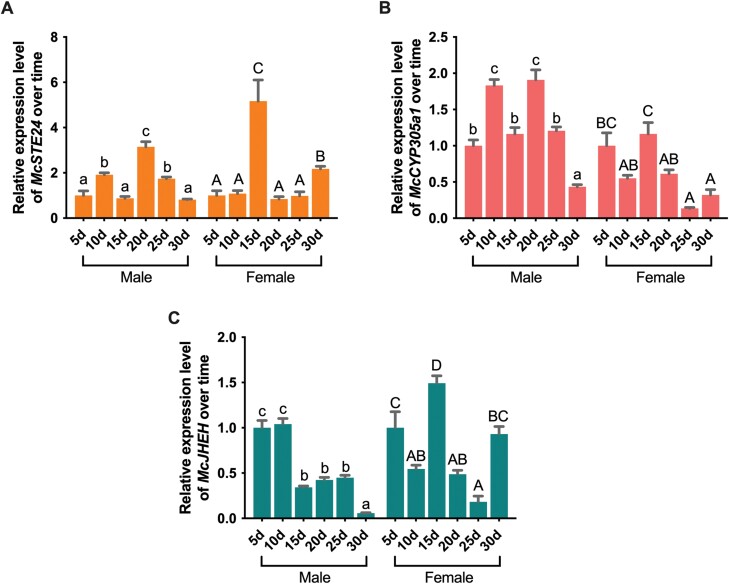
Time-specific relative expression patterns of *McSTE24*, *McCYP305a1*, and *McJHEH* in male and female *M. cichorii* adult beetles. Significant day effects are indicated using superscripts: a, b, c, and d for male groups and A, B, C, and D for female groups; groups that have the same letter did not differ significantly, and groups with a different letter differed significantly (*P* < 0.05). Values are means ± SEM. (*n* = 3). The expressions of different days were normalized to the expression at 5d in both males and females.

To investigate whether the expression patterns of 3 genes during development are time- and/or sex-specific, we performed RT-qPCR at different stages after emergence in both sexes. In males, 3 genes were highly expressed or showed a gradually significant increase in expressions at 5–10 days after eclosion, and subsequently declined significantly at day 15, then dramatically increased again at day 20 and/or 25, but significantly decreased thereafter at day 30. In females, the expression levels of the 3 genes were low or had a decreasing trend at 5–10 days after eclosion, followed by a significant increase at day 15, then significantly declined at day 20–25, but eventually increased again at 30 days (Fig. 2A–C). These results indicate that the expression patterns of 3 genes are different in males and females. Furthermore, the expression profiles from days 15–30 align with the trend of the cantharidin titer changes observed in males but not in females (Wang 2008).

**Fig. 3.**
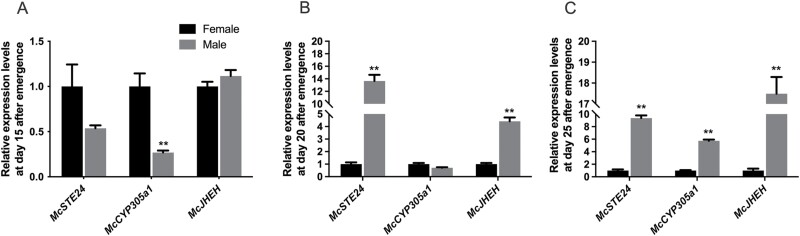
Relative expressions of *McSTE24*, *McCYP305a1*, and *McJHEH* in male adults compare with female adults at 15, 20, and 25 days after emergence. ** represents significant differences (*P* < 0.01, *n* = 3) between males and females. Values are means ± SEM. The expressions in males were normalized to the expression in females on different days.

The expression patterns at specific stages were also compared between male and female groups. There are no significant differences in the expression of *McSTE24* and *McJHEH* between males and females at day 15; for *McCYP305a1*, the expressions in males at that stage were even significantly lower than in females (*P* < 0.0001), indicating that these 3 genes expressed relatively low at the stage before peaking on cantharidin biosynthesis in males (Fig. 3A). However, the expressions of 3 genes increased sharply during the peak cantharidin biosynthesis stage (day 20–25) in males. The expressions of *McSTE24* and *McJHEH* significantly upregulated 13.6/9.3- and 4.4/17.5-fold in males than in females at day 20 and 25. Interestingly, at day 20, the expression of *McCYP305a1* had no significant difference between males and females, but it rose steeply at day 25 and significantly increased about 6-fold higher levels than its expressions in the females (Fig. 3B and C).

### *McSTE24, McCYP305a1*, and *McJHEH* Transcriptions Were Effectively Knocked Down by RNAi

**Fig. 4.**
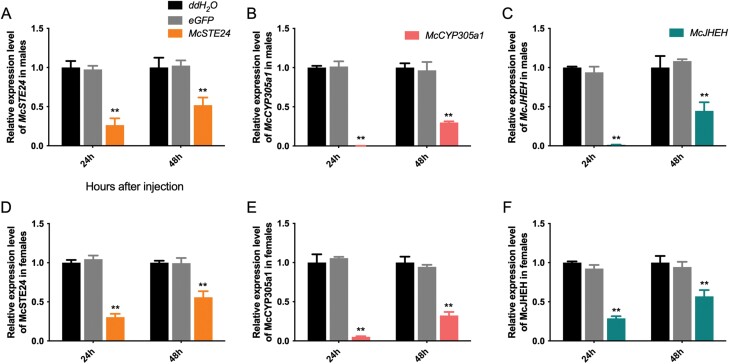
Expressions of *McSTE24*, *McCYP305a1*, and *McJHEH* in males and females at 24 h and 48 h after dsRNA injection. A–C) Expressions in males; D–F) Expressions in females. ** represents significant difference (*P* < 0.01, *n* = 3) between groups. Values are means ± SEM. Expressions of eGFP and dsRNA injected groups were normalized to ddH_2_O groups in both sexes.

RNAi efficacy was determined with RT-qPCR after the gene-specific dsRNAs were injected. Significant reductions in the transcript levels of *McSTE24*, *McCYP305a1*, and *McJHEH* were observed in both sexes. The reductions in the expression levels of *McSTE24*, *McCYP305a1*, and *McJHEH* were 73.56%, 99.89%, and 98.63%, respectively, in males at 24 h postinjection when compared to the ddH_2_O group. However, at 48 h postinjection, their expression was only reduced by 48%, 70%, and 55.31%, respectively (Fig. 4A–C). Similar to the males, the knockdown efficiencies of *McSTE24*, *McCYP305a1*, and *McJHEH* in females at 24 h post-injection were 69.6%, 94.79%, and 71.16%, respectively, when compared to the ddH_2_O group, whereas at 48 h postinjection, the interference efficiencies were 44.15%, 67.52%, and 43.06%, respectively (Fig. 4D–F).

We therefore conclude that the transcriptions of 3 genes were effectively knocked down by RNAi, and the most suitable sample collection time for the analyses of cantharidin content was 24h post-injection.

### *McSTE24, McCYP305a1* and *McJHEH* Play Critical Roles in Cantharidin Biosynthesis

**Fig. 5.**
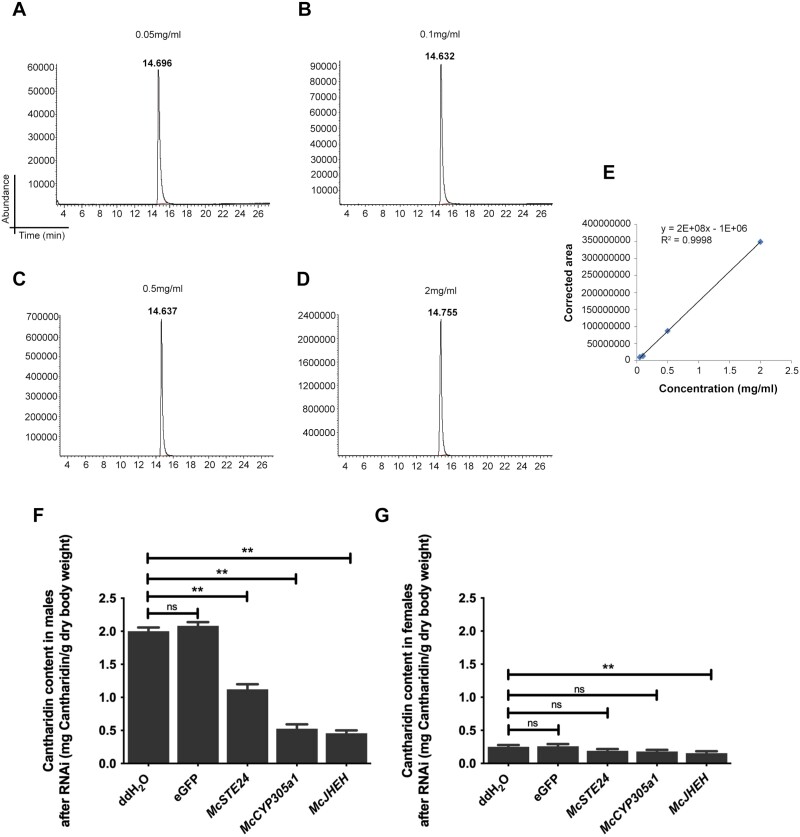
Cantharidin content in beetles after knockdown of *McSTE24*, *McCYP305a1*, and *McJHEH*. A–D) GC-MS spectra of standard cantharidin samples at the concentration of 0.05, 0.1, 0.5, 2 mg/ml, the *x*-axis indicates the peak retention time, the abundance in *y*-axis indicates the relative quantity of cantharidin; E) Standard curve based on the corrected area from the cantharidin standard samples at different concentrations (y = 2e + 08x—1e + 06, R^2^ = 0.9998); Cantharidin content (mg/g) after knockdown of each gene in F) males and G) females, normalized by dried beetle masses. ** represents significant differences (*P* < 0.01, respectively, *n* = 3) between each experimental group and controls. ns represents no significant differences between groups. Values are means ± SEM.

To investigate the roles of the 3 genes in cantharidin biosynthesis (the capacity of cantharidin synthesis), the cantharidin contents in males and females were determined at the peak of mass cantharidin biosynthesis after RNAi. A standard curve was plotted based on the corrected area from the cantharidin standard samples of 0.05, 0.1, 0.5, 2 mg/ml. Cantharidin content of each sample was then calculated based on the standard curve *y* = 2e + 08*x* −1e + 06, and normalized by the dried beetle body masses (g) (Fig. 5A–E). Compared with the control groups (ddH_2_O, 2 mg cantharidin/dry body weight g; *eGFP*, 2.082 mg/g), cantharidin production was significantly reduced in males 24 h after the *McSTE24*-*, McCYP305a1*-, and *McJHEH*-dsRNA treatments (cantharidin content after knockdown were 1.121 mg/g, 0.527 mg/g, and 0.458 mg/g in 3 groups, respectively, *P* < 0.0001 for all 3 comparisons) (Fig. 5F). The reductions in cantharidin in males were larger after *McCYP305a1* and *McJHEH* silencing than after *McSTE24* silencing, which may be because of the differences in RNAi efficiency (the efficiency of *McSTE24* was the lowest compared of the other 2 genes). In females, the cantharidin contents was significantly reduced to 0.155 mg/g at 24 h post-injection compared with the controls (ddH_2_O, 0.25 mg/g; *eGFP*, 0.26 mg/g) only after *McJHEH* was knockdown (Fig. 5G), suggesting that only *McJHEH* participate in cantharidin biosynthesis in females. Because females were housed separated from males after emergence, these results also indicated that females could self-synthesize a small amount of cantharidin, independent of receiving from males during mating.

### *McCYP305a1* Regulates *In Vivo* Cantharidin Synthesis Collaboratively With *McSTE24*, Whereas *McJHEH* Acts Independently, Separate From Either *McSTE24* or *McCYP305a1* in Males

The expression levels of the corresponding downstream genes were assessed with RT-qPCR at 24 h and 48 h after the injection of *McSTE24*- or *McCYP305a1*-dsRNA.

**Fig. 6.**
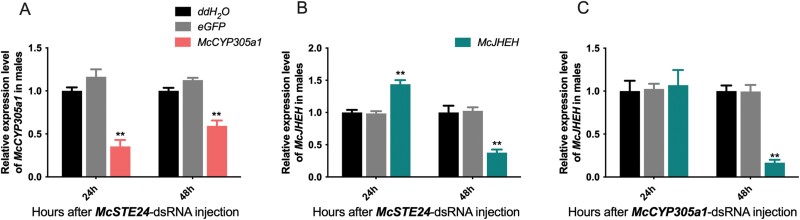
Expressions of downstream genes in males at 24 and 48 h after knockdown of *McSTE24* and *McCYP305a1*. A and B) Expressions of downstream genes *McCYP305a1* and *McJHEH* after knockdown of *McSTE24*; C) Expressions of downstream gene *McJHEH* after knockdown of *McCYP305a1*. ** represents significant difference (*P* < 0.01, *n* = 3) between groups. Values are means ± SEM. Expressions of eGFP and other dsRNA injected groups were normalized to ddH_2_O injected groups in both sexes.

After knockdown of *McSTE24*, downstream gene *McCYP305a1* was significantly downregulated at both 24 h and 48 h in males by 2.9- and 1.7 fold (*P* = 0.0015 and 0.0004). Interestingly, another downstream gene, *McJHEH*, was significantly upregulated at 24 h (*P* = 0.0016) and then downregulated at 48h (*P* = 0.0025) compared with the controls (Fig. 6A and B).

After knockdown of *McCYP305a1*, downstream gene *McJHEH* did not express significantly different between groups at 24 h (*P* = 0.9224) in males, and similarly, was then significantly downregulated in the experimental groups at 48 h (*P* = 0.0002) (Fig. 6C). These results suggested that *McCYP305a1* regulates cantharidin biosynthesis in a *McSTE24*-dependent manner while *McJHEH* regulates independently from both *McSTE24* and *McCYP305a1.*

## Discussion

In this study, we report cloning and functional analysis of 3 genes, *STE24*, *CYP305a1*, and *JHEH*, from the blister beetle *M. cichorii*. The cDNA sequences and deduced amino acid sequences of the 3 genes show high homologous degrees with the *STE24*, *CYP305a1*, and *JHEH* genes and proteins identified in other insects. The deduced protein sequences also share important structural features with the STE24, CYP305a1, and JHEH proteins of other insect species.

In male adult beetles, knockdown either of *McSTE24*, *McCYP305a1*, and *McJHEH* affected their capacity for cantharidin production. There was debate in the community about whether female blister beetles could synthesis cantharidin or not. Studies in the 20th century suggested that females do not synthesis cantharidin; they could only obtain it from males during copulation (Sierra et al. 1976, Holz 2015). Whereas there is more recent evidence indicating that females may also be able to synthesize cantharidin independently from the males’ nuptial transfer (Nikbakhtzadeh 2012, Carolina 2017). Supporting the latter idea, we demonstrated that female *M. cichorii* could self-synthesize very small amounts of cantharidin and the knockdown of *McJHEH* will impact such capacity, indicating that *McJHEH* participates in the cantharidin biosynthesis in females. Given the slight increase in cantharidin content in females from day 0 to day 10, followed by a decrease from day 15 to day 25 (Supplementary Table S1), it is evident that during the declining phase of cantharidin biosynthesis in female adults (day 15 to 25), the knockdown of *McSTE24* and *McCYP305a1*, 2 genes located upstream of the actionable cantharidin biosynthesis pathway, may still impact female cantharidin biosynthesis, albeit exerting subtle and undetectable effects on significant gene expression alterations. Conducting future RNAi experiments that include day 0 to 10 would be advantageous for dissecting the dynamic mechanisms underlying cantharidin biosynthesis in females.

Since knockdown of *McCYP305a1* (terminal gene contributes to JH synthesis in *M.cichorii*) and *McJHEH* (contribute to JH degradation) significantly reduced the *in vivo* cantharidin production in males, our results further demonstrated that cantharidin biosynthesis is associated with JH, coherent with the previous hypothesis (Carrel et al. 1986a, Mccormick and Carrel 1987, Huang et al. 2016) as well as recent evidence in other blister beetle species (Lü et al. 2016, Jiang et al. 2017a). A recent study (Liu et al. 2019) in *Ericerus pela* indicated that terpene compound genes are closely related to JH synthesis. In line with our results showing that *McSTE24* knockdown could suppress the expression of *McCYP305a1* (contribute to JH synthesis). Surprisingly, inhibition of *McSTE24* expression could somehow increase *McJHEH* (contribute to JH degradation) expression at 24 h after knockdown, but it decreased subsequently at 48 h after *McSTE24* knockdown. Similarly, after *McCYP305a1* knockdown, the expression of *McJHEH* also showed a delayed downregulation as described above, suggesting that *McCYP305a1* collaborates with its upstream gene *McSTE24* in cantharidin biosynthesis, whereas *McJHEH* acts in such a process independently from both upstream genes, *McSTE24* and *McCYP305a1.* These results indicated that after *McSTE24* or *McCYP305a1* knockdown for 24 h may reduce the accumulation of the JH precursor, which will temporarily increase or keep the expression level of *McJHEH* to maintain the JH degradation or cantharidin synthesis related biological process. After *McSTE24* or *McCYP305a1* knockdown for 48 h, with the continuous reduction of the precursor for JH metabolism, the demand for the JHEH enzyme to degrade JH is therefore also reduced, eventually resulting in the suppression of the *McJHEH* gene expression. * McSTE24*/*McCYP305a1* and *McJHEH* might constitute a compensating system to regulate cantharidin titer in male *M. cichorii*. These findings also indicated that the terpenoid backbone pathway may regulate cantharidin biosynthesis in conjunction with JH synthesis pathways but independently of JH degradation pathways in *M. cichorii*.

Male adults begin their gonadal development after emergence, and the mating with females usually occurs at 20–25 days after emergence in *M.cichorii*. During mating, the males will pass most of their cantharidin reserves to the females (Sierra et al. 1976). As a result, the males need to synthesize a large amount of cantharidin during or after the age of mating (at approximately 20–25 days) to maintain their own cantharidin levels or to prepare for possible further repeated copulation (Wang 2008, Garófalo 2011, Ghoneim 2012). Coherent with the cantharidin titer changes at the corresponding phase, we found that the expression levels of *McSTE24*, *McCYP305a1*, and *McJHEH* were significantly upregulated at day 20 or 25 in the males compared with day 15 (Supplementary Table S1). Interestingly, the high expressions of the *McSTE24* and *McCYP305a1* in males at day 10 were not consistent with the low cantharidin titer at the same stage. (Supplementary Table S1). Since cantharidin biosynthesis is related to JH (Mccormick et al. 1986, Mccormick and Carrel 1987, Huang et al. 2016), *McSTE24*, *McCYP305a1*, and *McJHEH* at this stage may also participate in JH biosynthesis or degradation in male *M. cichorii*. Given that JH regulates development and reproductive maturation in insects (Goodman and Granger 2005), these genes may exert more dominant roles in physiological processes such as gonadal development during the early stages after emergence in males. As gonadal development is typically completed by day 20 or 25, these genes may predominantly contribute to cantharidin biosynthesis during this period. However, the reasons behind the significantly higher expression of *McJHEH* at day 5 and 10 compared to other time points remain unknown and warrant further investigation. Interestingly, at day 20, the expression of *McCYP305a1* had no significant difference between males and females, but it increased significantly to about 6-fold higher levels at day 25 compared to females. This might explain why *McCYP305a1* did not differ between males and females in the previous transcriptome library (Huang et al. 2016). It was likely because the measured samples were pooled across 20–25 days instead of being separated at day 20 and 25, therefore unable to detect the dramatic switch in gene expression over 20–25 days in males. Together, our results indicated that the 3 genes impact differently on males and females as well as on their different developmental stages. The expression pattern of *McSTE24* and *McCYP305a1* were similar in males across stages but are different from *McJHEH*. This further supports the findings that *McSTE24* and *McCYP305a1* work collaboratively in cantharidin biosynthesis, whereas *McJHEH* functions independently. While in females, *McCYP305a1* and *McJHEH* has similar expression patterns across different stages, which are distinct from *McSTE24*. Despite adult female *M. cichorii* could synthesize a small amount of cantharidin and *McJHEH* might act in such process, the time-specific expression changes of 3 genes are not consistent with their cantharidin titer changes (Supplementary Table S1), indicating that these 3 genes may play dominant roles in other physiological activities such as reproductive development in females.

Future functional studies on cantharidin biosynthesis stand to benefit from the increasing availability of genome sequencing data across various blister beetle species (Wu et al. 2018, Guan et al. 2020, Zhou et al. 2023, Riccieri et al. 2024), which will broaden our understanding of the genes and pathways involved. Another intriguing avenue for future research would be to explore dual RNAi approaches targeting multiple key genes within pivotal pathways simultaneously to comprehensively understand the synergistic effects of different genes on cantharidin biosynthesis.

In conclusion, we found *McSTE24*, *McCYP305a1*, and *McJHEH* are essential in cantharidin biosynthesis in *M. cichorii*. Our results also revealed a novel role of *STE24* as an important gene act in cantharidin in vivo synthesis, which has not been addressed in any of the blister beetles. We also found that *McCYP305a1*, associated with JH biosynthesis, collaboratively regulates cantharidin synthesis with its upstream gene *McSTE24* from the terpenoid backbone pathway. Conversely, *McJHEH*, involved in JH degradation, appears to independently influence cantharidin biosynthesis, separate from the regulatory influence of either *McCYP305a1* or *McSTE24*. Furthermore, the expression patterns of the 3 genes were found to be both time- and sex-specific. Specifically, their expression correlated solely with cantharidin levels during the peak synthesis period (20–25 days old) in males but not at other developmental stages. Conversely, in females, the expression of these genes did not correlate with in vivo cantharidin levels across any developmental stage, suggesting their potential involvement in other biological processes specific to females. Our study expanded the current knowledge of in vivo cantharidin synthesis and would be beneficial for the scientific community to further understand the molecular mechanisms of cantharidin biosynthesis.

## Supplementary Material

ieae070_suppl_Supplementary_Tables_S2

ieae070_suppl_Supplementary_Tables_S3

ieae070_suppl_Supplementary_Tables_S4

ieae070_suppl_Supplementary_Figures_S1

ieae070_suppl_Supplementary_Figures_S2

ieae070_suppl_Supplementary_Tables_S1
