# Supplementary material for: Functional studies of McSTE24, McCYP305a1, and McJHEH, three essential genes act in cantharidin biosynthesis in the blister beetle (Coleoptera: Meloidae)
Source: J Insect Sci. 2024 Jul 11;24(4):4. doi: 10.1093/jisesa/ieae070 (PMC11237990; doi:10.1093/jisesa/ieae070)
Supplement: ieae070_suppl_Supplementary_Tables_S2 [file ieae070_suppl_supplementary_tables_s2.pdf]

Suppl. Table S2 Primers for gene cloning

Primer name	Primer sequences (forward and reverse)
STE24check-L	5'-GGAATCGTACTTATCGAGTCGTCAA-3'
STE24check-R	5'-TTAATAACGGCGGATGCGAATGAT-3'
FP1	5'-GTAATACGACTCACTATAGGGCACGCGTGGTNTCGASTWTSGWGTT-3'
FP2	5'-GTAATACGACTCACTATAGGGCACGCGTGGTNGTCGASWGANAWGAA-3'
FP3	5'-GTAATACGACTCACTATAGGGCACGCGTGGTWGTGNAGWANCANAGA-3'
FP4	5'-GTAATACGACTCACTATAGGGCACGCGTGGTAGWGNAGWANCAWAGG-3'
FP5	5'-GTAATACGACTCACTATAGGGCACGCGTGGTNGTAWAASGTNTSCAA-3'
FP6	5'-GTAATACGACTCACTATAGGGCACGCGTGGTNGACGASWGANAWGAC-3'
FP7	5'-GTAATACGACTCACTATAGGGCACGCGTGGTNGACGASWGANAWGAA-3'
FP8	5'-GTAATACGACTCACTATAGGGCACGCGTGGTGTNCGASWCANAWGTT-3'
FP9	5'-GTAATACGACTCACTATAGGGCACGCGTGGTNCAGCTWSCTNTSCTT-3'
FSP1	5'-GTAATACGACTCACTATAGGGC-3'
FSP2	TATAGGGCACGCGTGGT-3'
STE24SP1	5'-TGAGCAACCATAAATAGAGGAAG-3'
STE24SP2	5'-TATAAGTACCGAAATTGTGCC-3'
STE24SP3	5'-GTCGGTACTTCTCGTATTTCTTTC-3'
CYP305a1SP1-L	5'-TTTGGCCAGTTTCTTCAATTCGGGT-3'
CYP305a1SP2-L	5'-AGATTGCCAATTAGCGGTAACCAT-3'
CYP305a1SP3-L	5'-TCGTTGGTCTTTCGATGTCTTTCA-3'
CYP305a1SP1-R	5'-GGCAAACTGTTAAGTCCAGATAGAT-3'
CYP305a1SP2-R	5'-TTCGGTCTAGGTAGACGTCGGTGTT-3'
CYP305a1SP3-R	5'-AAGCCGTTACCTGGCATCACTCTAT-3'
JHEHSP1	5'-TTGATCAGTCCACCAAACATCCTTC-3'
JHEHSP2	5'-TTTGGGAATTTGCGGGATTTCTGTT-3'
JHEHSP3	5'-CCCACCATTGTCGTAAAAATTCCAC-3'
3'RACE primer	5'-TACCGTCGTTCCACTAGTGATTT-3'
STE24GSP	5'-GGTTTCCCGATTTACGATGA-3'
JHEHGSP	5'-AACTATTCCAGCAGCGTATG-3'
STE24-ORF-L	5'-CGCTTTGTTGATTAGAAAATCGCTT-3'
STE24-ORF-R	5'-AAAATCAAGATTGATTTCGTCTGATTTGG-3'
CYP305a1-ORF-L	5'-AATAGATTCGTCAAAGTGAGCGTG-3'
CYP305a1-ORF-R	5'-TTATTTTATTGGATTCTTTCAG-3'

Primer name	Primer sequences (forward and reverse)
JHEH-ORF-L	5’-AACCTGCTTCGTAATTGTGA-3’
JHEH-ORF-R	5’-TTAATAGCCATACATGTAGTACCGTT-3’

Note: V=A, G, or C; N = A , G , C or T
