# Supplementary material for: Functional studies of McSTE24, McCYP305a1, and McJHEH, three essential genes act in cantharidin biosynthesis in the blister beetle (Coleoptera: Meloidae)
Source: J Insect Sci. 2024 Jul 11;24(4):4. doi: 10.1093/jisesa/ieae070 (PMC11237990; doi:10.1093/jisesa/ieae070)
Supplement: ieae070_suppl_Supplementary_Tables_S3 [file ieae070_suppl_supplementary_tables_s3.pdf]

Suppl. Table S3 Primers used for expression pattern analysis

Gene	Primer sequences (forward and reverse)
RPL22e	5'-AGGCTTGAAGAAGAAGAAG-3'
	5'-ATGTTGGCTGGATTTACC-3'
UBE3A	5'-TTAGTGAATGCTCTAGTAAC-3'
	5'-CGGTAATGCTGTCTCTAA-3'
STE24	5'-CCACACAATTGAATTTCCCA-3'
	5'-CCACACAAATAGGCGTTACTG-3'
CYP305a1	5'-GGCGACTTGTGGTCTATTCA-3'
	5'-CTTCACCATCAATTCCATCG-3'
JHEH	5'-CGAACATGTGTTATGCGACA-3'
	5'-CAAGAAGACGATTGGCGTAA-3'
