# Supplementary material for: Functional studies of McSTE24, McCYP305a1, and McJHEH, three essential genes act in cantharidin biosynthesis in the blister beetle (Coleoptera: Meloidae)
Source: J Insect Sci. 2024 Jul 11;24(4):4. doi: 10.1093/jisesa/ieae070 (PMC11237990; doi:10.1093/jisesa/ieae070)
Supplement: ieae070_suppl_Supplementary_Tables_S4 [file ieae070_suppl_supplementary_tables_s4.pdf]

Suppl. Table S4 Primers used for dsRNA synthesis

Gene	Primer sequences (forward and reverse)
eGFP	5'-TAATACGACTCACTATAGGGACGTAAACGGCCACAAGTTC-3'
	5'-TAATACGACTCACTATAGGGTGCTCAGGTAGTGGTTGTCG-3'
STE24	5'-TAATACGACTCACTATAGGGAAGGTTCGAAACGTTCATCG-3'
	5'-TAATACGACTCACTATAGGGGCCAGGCCGAATATAAATCA-3'
CYP305a1	5'-TAATACGACTCACTATAGGGTCCCGACAACTTCTTCATCC-3'
	5'-TAATACGACTCACTATAGGGGCCGGCGATAAACAAATCTA-3'
JHEH	5'-TAATACGACTCACTATAGGGTTTTGGCACTTGTTGTGGAA-3'
	5'-TAATACGACTCACTATAGGGACTTCGAAAATGAACCGACG-3'

Note: The sequence of T7 promoter region is TAATACGACTCACTATAGGG
