# Supplementary material for: Functional studies of McSTE24, McCYP305a1, and McJHEH, three essential genes act in cantharidin biosynthesis in the blister beetle (Coleoptera: Meloidae)
Source: J Insect Sci. 2024 Jul 11;24(4):4. doi: 10.1093/jisesa/ieae070 (PMC11237990; doi:10.1093/jisesa/ieae070)
Supplement: ieae070_suppl_Supplementary_Figures_S1 [file ieae070_suppl_supplementary_figures_s1.pdf]

(A)

CGCTTTGTTGATTAGAAAATCGCT
TAGAAGAGCTTAATAACTAGACTGAATTTCATAAATTATCGACA

1 atgaacgtgccagaagaactaataataagatatgccattctgtcg
M N V P E E L I I R Y A I L S
46 tttacatgggcggaatatttatgggaatcgtacttatcgagtcgt
F T W A E Y L W E S Y L S S R
91 caatatcgtaaagtgaaagaaatacgagaagtaccgacaatatta
Q Y R K V K E I R E V P T I L
136 gaaggtacaataacacaagaaatgttcgataaagctcgattatac
E G T I T Q E M F D K A R L Y
181 aatttagccaaacttcaatttggttttattatcggcacaatttcg
N L A K L Q F G F I I G T I S
226 gtacttatatcgacgattgttattacttgtaatatttttttatta
V L I S T I V I T C N I F L L
271 atttggaatcttgcaatgtcaattcgaattgttgatagtgaaatt
I W N L A M S I R I V D S E I
316 ttaacaagttgcatatgggtatcgattttattaacaatatcagcg
L T S C I W V S I L L T I S A
361 ataattgaattaccattaaccatctattatactttggtttagag
I I E L P L T I Y Y T F G L E
406 gagaaattcggtttcaataagcagacagtcttctttttcatttgg
E K F G F N K Q T V F F F I W
451 gataacacgaaacaattcatgttaattcacatcttctcatggata
D N T K Q F M L I H I F S W I
496 ataacctcattgattatcgttctaattaaaagtagtggtgatttt
I T S L I I V L I K S S G D F
541 ttcttcctctatttatggttgctcatttgtatcatgataattgtc
F F L Y L W L L I C I M I I V
586 ttgtgtttttatatccatgggtaattgcgccagttttcgataaa
L C F L Y P W V I A P V F D K
631 ttcgtaccgttaccggaaggtgaattgcgtacagaaatcgagaat
F V P L P E G E L R T E I E N
676 ttagccacacgattaaatttcccattaaatcaactgtatgtagta
L A T R L N F P L N Q L Y V V
721 gaaggttcgaaacgttcatcgcacagtaacgcctatttgtgtggt
E G S K R S S H S N A Y L C G
766 ttatttaaaacgaaacgtattgtcctatttgatacgttattggcg
L F K T K R I V L F D T L L A
811 aaaagagatggtgagagtgtttataagaatgatgagattttggcg
K R D G E S V Y K N D E I L A
856 attctaacgcacgaattaggccattggaaatacaatcatattatc
I L T H E L G H W K Y N H I I
901 aaaaaaatgatcttcattcaaatgaatctattactattatttatt
K K M I F I Q M N L L L L F I
946 gctttttcgtttctatttaaatatccaccaatctattatgctttc
A F S F L F K Y P P I Y Y A F
991 ggttttatgaccaacaaccagttctaatcggtttaattattcta
G F Y D Q Q P V L I G L I I L
1036 caatatctaatgataccgtacaatatttgtcgagtttcttgatg
Q Y L M I P Y N I L S S F L M
1081 aattatttatcgcgtaaattcgaattacaagctgatttatttgct
N Y L S R K F E L Q A D L F A
1126 gtagaattaaatcgaggtgaaccattaatcagagctttaatacaa
V E L N R G E P L I R A L I Q
1171 atgaataaagacaatttaggtttcccgatttacgatgatttatat
M N K D N L G F P I Y D D L Y
1216 tcggcctggcatcattcgcatccaccgttattaaaacgaatcgct
S A W H H S H P P L L K R I A
1261 atcctcaaaagagctgcccaaatcagacgaaatcaatcttga 1302
I L K R A A Q I R R N Q S *

TTTTATAAATATTCGTGTATTTGTGATAAAATTTGTTA**AATAAA**C
GATTCTATTTGAAAAAAAAAAAA

(B)

AATAGATTCGTCAAAGTGAGCGTGATTCAC
ATCTTCCGCACCAATTTATCACGATAAACGTAACTGAAATTTCT
CGAAAATATGCAATTTTAACTAAAGAAACTGTAGAAAATAAAA

```
   1 atgtttttatttataatttttatcggtttgattgtgatttatttg
     M  F  L  F  I  I  F  I  G  L  I  V  I  Y  L
  46 ttgaaagacatcgaaagaccaacgaatttcccccccaggtccgcga
     L  K  D  I  E  R  P  T  N  F  P  P  G  P  R
  91 tggttaccgctaattggcaatctacccgaattgaagaaactggcc
     W  L  P  L  I  G  N  L  P  E  L  K  K  L  A
 136 aaatctttaggcggccaacatttagcccttagtgaattgtcgaga
     K  S  L  G  G  Q  H  L  A  L  S  E  L  S  R
 181 atctacaatacgaacgtgttaggcttgaaattgggtagcgattat
     I  Y  N  T  N  V  L  G  L  K  L  G  S  D  Y
 226 gttgtcgttgtattctcctacaatacggtccgccaggtgctgact
     V  V  V  V  F  S  Y  N  T  V  R  Q  V  L  T
 271 agagaggagtttgaagggcgtcccgacaacttcttcatccgattg
     R  E  E  F  E  G  R  P  D  N  F  F  I  R  L
 316 cgctgcatgggaatcaggcgaggtgtaacgtgtacagacggcgac
     R  C  M  G  I  R  R  G  V  T  C  T  D  G  D
 361 ttgtggtctattcaacgaaatttcgtggtccgtcatttgcgcaat
     L  W  S  I  Q  R  N  F  V  V  R  H  L  R  N
 406 ttgggctttggtaaaaagccgatggaattgatggtgaagaacgaa
     L  G  F  G  K  K  P  M  E  L  M  V  K  N  E
 451 atagaatcggttttaacaacattgcaacaagacaatattcatatt
     I  E  S  V  L  T  T  L  Q  Q  D  N  I  H  I
 496 ggtaaaaccctagcaccggtcgtaataaacatattatggatgtta
     G  K  T  L  A  P  V  V  I  N  I  L  W  M  L
 541 ataaccggcaatcaattgagcacaaatcatcaacaattggatcgt
     I  T  G  N  Q  L  S  T  N  H  Q  Q  L  D  R
 586 ttgttggatttatttgagttgcgttcgaaggctttcgatatgtcc
     L  L  D  L  F  E  L  R  S  K  A  F  D  M  S
 631 ggcggtactttaacgcaatacccatggctgagatttgtagcacca
     G  G  T  L  T  Q  Y  P  W  L  R  F  V  A  P
 676 gaacgatctggttataatttaatacaaacgatcaataaacaatta
     E  R  S  G  Y  N  L  I  Q  T  I  N  K  Q  L
 721 gacgagttatttatggagacgatcaatgaacatcaacgaaattgg
     D  E  L  F  M  E  T  I  N  E  H  Q  R  N  W
 766 aatgaaaatcgagatgatgatttaatatactcgtatataactgaa
     N  E  N  R  D  D  D  L  I  Y  S  Y  I  T  E
 811 atgaaacagaataattgcaaggaaatattcacgtacgaacaatta
     M  K  Q  N  N  C  K  E  I  F  T  Y  E  Q  L
 856 gtgatggtttgtttagatttgtttatcgccggcacccagactact
     V  M  V  C  L  D  L  F  I  A  G  T  Q  T  T
 901 agtaatacgttgaatttcgcattcctgatgatgattttatatcca
     S  N  T  L  N  F  A  F  L  M  M  I  L  Y  P
 946 gaaatacaagaaaaagtccaccaagaaatcgaccaatttctcaac
     E  I  Q  E  K  V  H  Q  E  I  D  Q  F  L  N
 991 ggtgataatctcacgtattccgatcgccataaattaccgtacacg
     G  D  N  L  T  Y  S  D  R  H  K  L  P  Y  T
1036 gaggctgttttattagaagtggaacggtactgccatgtggtacca
     E  A  V  L  L  E  V  E  R  Y  C  H  V  V  P
1081 atctgtggccctagaagagtattgcgcgatacaattttggagggt
     I  C  G  P  R  R  V  L  R  D  T  I  L  E  G
1126 taccatatacctaaagatactacagttctaattagtttgtattcc
     Y  H  I  P  K  D  T  T  V  L  I  S  L  Y  S
1171 gtccataaggatcaagaacactggaaagatccggaagtattccgt
     V  H  K  D  Q  E  H  W  K  D  P  E  V  F  R
1216 cctgatcgatttctcgatagtacaggcaaactgttaagtccagat
     P  D  R  F  L  D  S  T  G  K  L  L  S  P  D
1261 agattaataccgttcggtctaggtagacgtcggtgtttaggcgaa
     R  L  I  P  F  G  L  G  R  R  R  C  L  G  E
1306 attctagctaaaacttgcatatttatgctattcgtggagattctc
     I  L  A  K  T  C  I  F  M  L  F  V  E  I  L
1351 cgaaaattcaaaattactcaaaaatccatcgatagaaaacctatt
     R  K  F  K  I  T  Q  K  S  I  D  R  K  P  I
1396 gaaaagccgttacctggcatcactctatcaccacagccgtaccgt
     E  K  P  L  P  G  I  T  L  S  P  Q  P  Y  R
1441 gctcagtttttagagcgccattctgaaagaatccaataa 1479
     A  Q  F  L  E  R  H  S  E  R  I  Q  *
```

AATAAGTTTTATTTTAAAAATTTCGT**AATAAA**TACCCTGGAGAC
AAAAAAAAAAAA

(C)

```
                               AACCTGCTTCGTAATTGTGATTTTTCA
   GATTTATAAATTTTTATAGATAATGGTTTTATCTCTGATAGTCTC
   AATCACGACATATTTGTATTATTTAACCGCGATTAACGTAAAATA
   1 atgtcggttgtattgtttctagttttagtaataattttggcactt
     M  S  V  V  L  F  L  V  L  V  I  I  L  A  L
  46 gttgtggaatttttacgacaatggtgggttaaaataacagaaatc
     V  V  E  F  L  R  Q  W  W  V  K  I  T  E  I
  91 ccgcaaattcccaaattgaaggatgtttggtggactgatcaagat
     P  Q  I  P  K  L  K  D  V  W  W  T  D  Q  D
 136 ccggctcaggaagactccacaatacagccgtttaaaatccatgtt
     P  A  Q  E  D  S  T  I  Q  P  F  K  I  H  V
 181 cccgatgaagctttagaagatctaaaaaatcgcctatcaaatgca
     P  D  E  A  L  E  D  L  K  N  R  L  S  N  A
 226 aagcctctaacccacccattggaaagcatacaacaccaatacggt
     K  P  L  T  H  P  L  E  S  I  Q  H  Q  Y  G
 271 atcaatacgaaattattaaatgaaattatcgaattttggcgtaca
     I  N  T  K  L  L  N  E  I  I  E  F  W  R  T
 316 aagtacaattggcgagaacgtgaagcattcctgaataaattccca
     K  Y  N  W  R  E  R  E  A  F  L  N  K  F  P
 361 caatatacagttaacgttcagggattacgcatccattatctccat
     Q  Y  T  V  N  V  Q  G  L  R  I  H  Y  L  H
 406 gtaaaaccaacagaaactgctgatttaaaagttgttccgattctt
     V  K  P  T  E  T  A  D  L  K  V  V  P  I  L
 451 ctgttacacggatggccgggctcaattcgggagttctatgaattg
     L  L  H  G  W  P  G  S  I  R  E  F  Y  E  L
 496 ataccgattttaacaaatccacaacctggacgtcggttcatttttc
     I  P  I  L  T  N  P  Q  P  G  R  R  F  I  F
 541 gaagtaatcgccccttcattacccggctttggtttctcccaagcc
     E  V  I  A  P  S  L  P  G  F  G  F  S  Q  A
 586 gccacaaaacccggtctaggtgctgtacagttagctgtagttttc
     A  T  K  P  G  L  G  A  V  Q  L  A  V  V  F
 631 aagaatttcatgcagaaattaggctttgaaaaatactacatacag
     K  N  F  M  Q  K  L  G  F  E  K  Y  Y  I  Q
 676 ggtggtgattgggggggcgattattgtccaacatatggcaacactc
     G  G  D  W  G  A  I  I  V  Q  H  M  A  T  L
 721 tatccggaacatatactcggtctacattcgaacatgtgttatgcg
     Y  P  E  H  I  L  G  L  H  S  N  M  C  Y  A
 766 acaagttttatgataacattaaaaaccttgttgttcagcttgaag
     T  S  F  M  I  T  L  K  T  L  L  F  S  L  K
 811 ccgtcatggttccttgaagatcgattcgttaaacgcctgtatcca
     P  S  W  F  L  E  D  R  F  V  K  R  L  Y  P
 856 ttgaaagaatattacgccaatcgtcttcttgagactggctacgtc
     L  K  E  Y  Y  A  N  R  L  L  E  T  G  Y  V
 901 cacttacaagcaacaaaaccagatacaatcggtgtgggtttggcg
     H  L  Q  A  T  K  P  D  T  I  G  V  G  L  A
 946 gattccccgatcggtttggctgcttacattctagaaaaattcatc
     D  S  P  I  G  L  A  A  Y  I  L  E  K  F  I
 991 acttggacaaatccggaatacaaaaatagttttgacggtggctta
     T  W  T  N  P  E  Y  K  N  S  F  D  G  G  L
1036 aacgagaaattctcatacgcaaatttattggataatgtcatgatt
     N  E  K  F  S  Y  A  N  L  L  D  N  V  M  I
1081 tattggataacaaattcgataacaacatcaatgcgattgtatgct
     Y  W  I  T  N  S  I  T  T  S  M  R  L  Y  A
1126 gaaactttcagtctaaaacagaaacgattaaatgttgataagatc
     E  T  F  S  L  K  Q  K  R  L  N  V  D  K  I
1171 cctataactattccagcagcgtatgcacgatttctcatgaaata
     P  I  T  I  P  A  A  Y  A  R  F  S  H  E  I
1216 atttatacacccagatgtttattggaggagaaattcaagaaatta
     I  Y  T  P  R  C  L  L  E  E  K  F  K  K  L
1261 ttgcatgaatcggattatgaaggtgggcattttgctgcattcgaa
     L  H  E  S  D  Y  E  G  G  H  F  A  A  F  E
1306 tgcccaaatatttggcgaatgatatttatgaagctgtcgccaga
     C  P  N  I  L  A  N  D  I  Y  E  A  V  A  R
1351 tttgaacggtactacatgtatggctattaa 1380
     F  E  R  Y  Y  M  Y  G  Y  *
   AAGAAAATTTTGATTTTATCACTTTGTAGAGAAAAACTTAAATAA
   ACGGATTCTATGATTGCAAAAAAAAAAAA
```

Suppl. Fig. S1. (A) Full length sequence of *McSTE24* with deduced amino acid sequence; (B) Full length sequence of *McCYP305a1* with deduced amino acid sequence; (C) Full length sequence of *McJHEH* with deduced amino acid sequence. The start codon (ATG), termination codon were highlight in red and * indicated termination codon, the polyadenylation signal (AATAAA) is in bold.
