# Supplementary material for: Functional studies of McSTE24, McCYP305a1, and McJHEH, three essential genes act in cantharidin biosynthesis in the blister beetle (Coleoptera: Meloidae)
Source: J Insect Sci. 2024 Jul 11;24(4):4. doi: 10.1093/jisesa/ieae070 (PMC11237990; doi:10.1093/jisesa/ieae070)
Supplement: ieae070_suppl_Supplementary_Figures_S2 [file ieae070_suppl_supplementary_figures_s2.pdf]

(A)

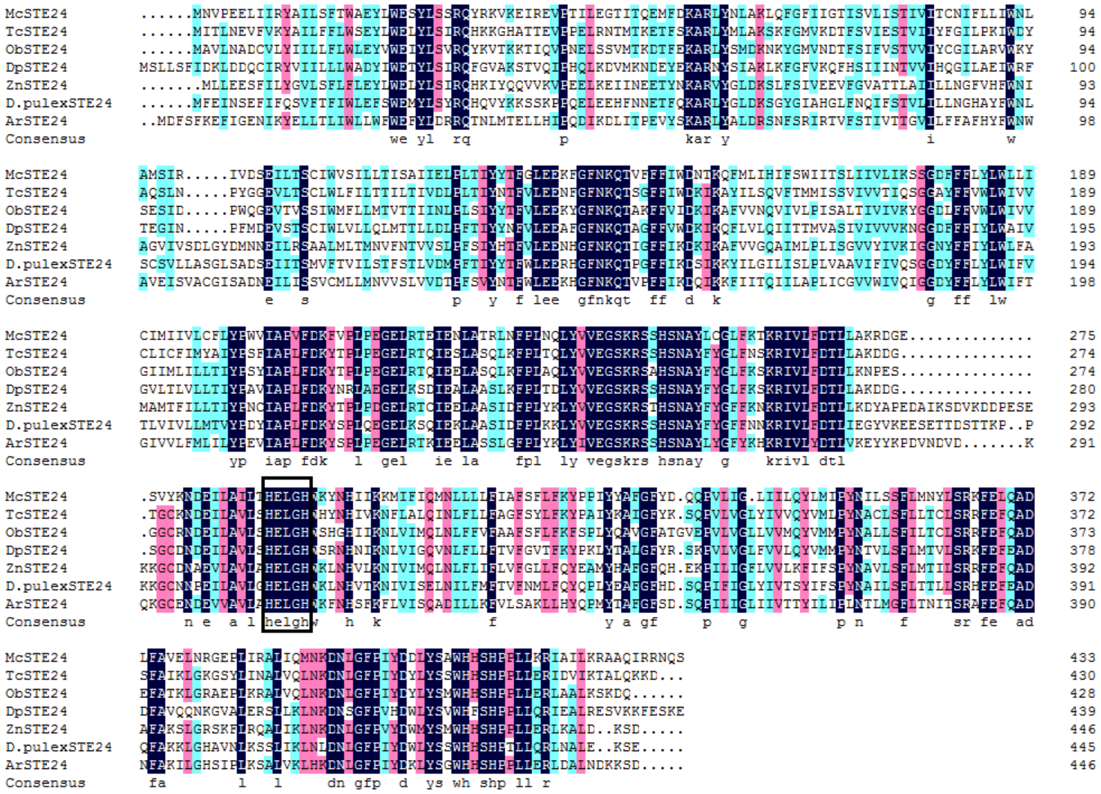
McSTE24
TcSTE24
ObSTE24
DpSTE24
ZnSTE24
D.pulexSTE24
ArSTE24
Consensus

(B)

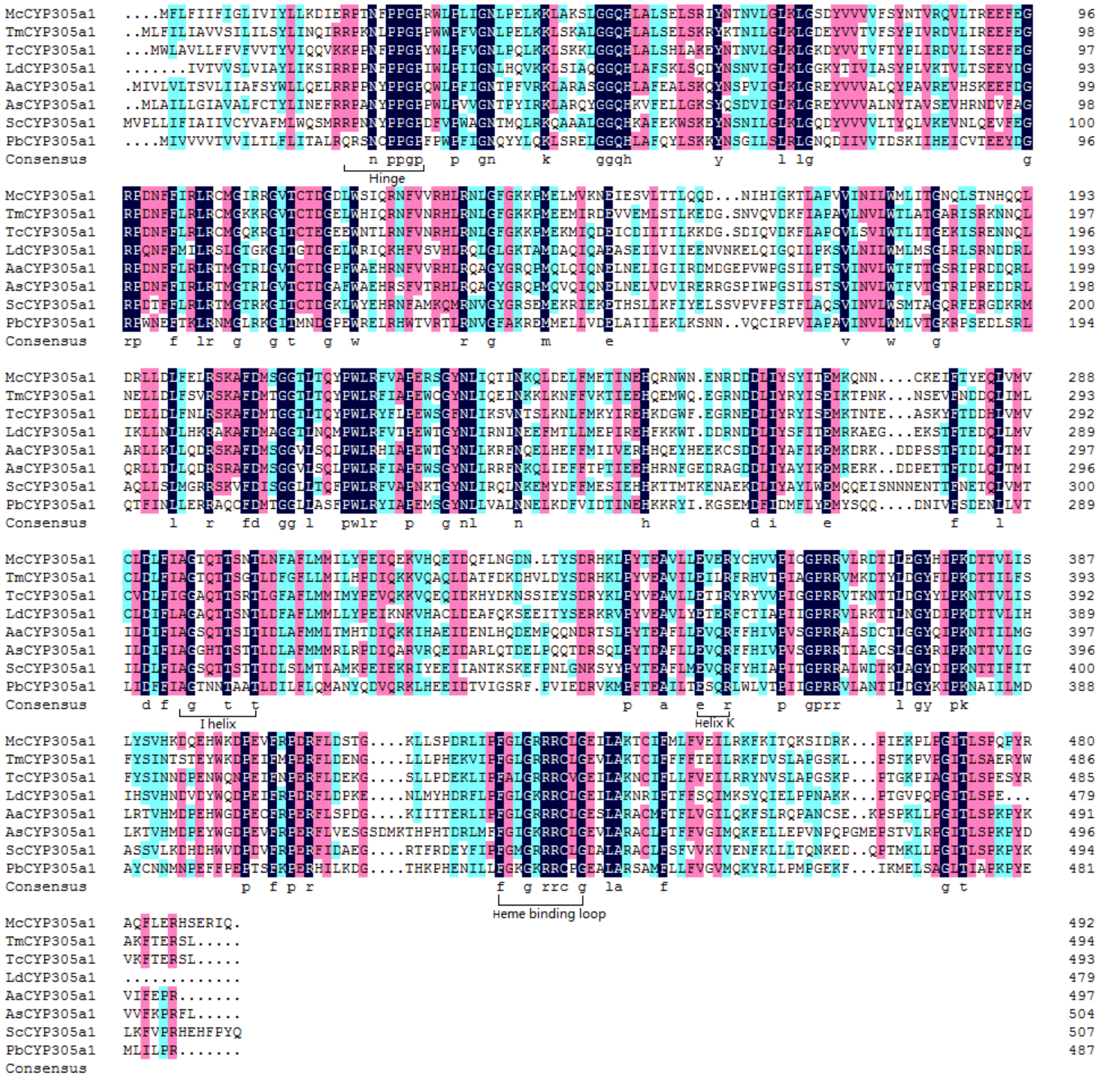
McCYP305a1
TmCYP305a1
TcCYP305a1
LdCYP305a1
AaCYP305a1
AsCYP305a1
ScCYP305a1
PbCYP305a1
Consensus
Hinge
I helix
Helix K
Heme binding loop

(C)

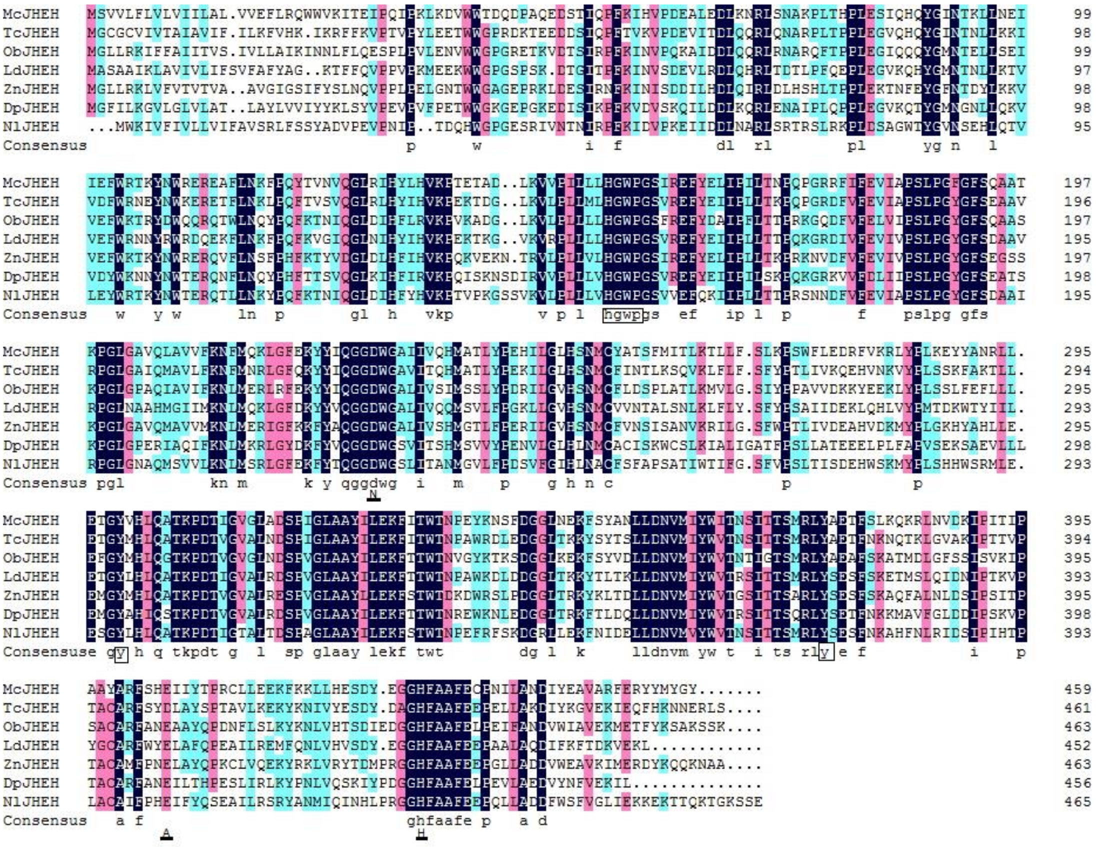

Suppl. Fig. S2. (A) Amino acid sequence multiple alignment of McSTE24 with several other species. TcSTE24 represents *Tribolium castaneum* (XP_968656.1), ObSTE24 represents *Oryctes borbonicus* (KRT82335.1), DpSTE24 represents *Dendroctonus ponderosae* (ENN82041.1), ZnSTE24 represents *Zootermopsis nevadensis* (KDR22915.1), D.pulexSTE24 represents *Daphnia pulex* (EFX72100.1), ArSTE24 represents *Athalia rosae* (XP_012250805.1). The black box indicates an HEXXH Zn^{2+}-metalloprotease signature; (B) Amino acid sequence multiple alignment of McCYP305a1 with several other insect CYP305a1. TmCYP305a1 represents *Tenebrio molitor* (AKZ17688.1), TcCYP305a1 represents *T.castaneum* (XP_970235.1), LdCYP305a1 represents *Leptinotarsa decemlineata* (AGT57832.1), AaCYP305a1 represents *Aedes aegypti* (XP_001654598.1), AsCYP305a1 represents *Anopheles sinensis* (KFB45025.1), ScCYP305a1 represents *Stomoxys calcitrans* (XP_013107850.1), PbCYP305A1 represents *Pogonomyrmex barbatus* (XP_011646186.1). The motifs Hinge, I helix, Helix K, Heme binding loop are respectively indicated with their underline names below the sequence; (C) Amino acid sequence multiple alignment of McJHEH with several other insect JHEH. TcJHEH represents *T.castaneum* (EFA00568.1), ObJHEH represents *O. borbonicus* (KRT85408.1), LdJHEH represents *L. decemlineata* (AKF11871.1), ZnJHEH represents *Z. nevadensis* (KDR10172.1), DpJHEH represents *D. ponderosae* (AEE63126.1), NlJHEH represents *Neodiprion lecontei* (XP_015524910.1). The

conservative properties of amino acid residues are displayed by three marked patterns, black, red, blue, indicating the conservation passing from the highest to the lowest. The residues that form the potential catalytic triad of JHEH are underlined in black, indicated by N (nucleophile), A (acidic residue), and H (histidine), while those residues consisting of the potential oxyanion hole are indicated by black box.
