# Supplementary material for: Functional studies of McSTE24, McCYP305a1, and McJHEH, three essential genes act in cantharidin biosynthesis in the blister beetle (Coleoptera: Meloidae)
Source: J Insect Sci. 2024 Jul 11;24(4):4. doi: 10.1093/jisesa/ieae070 (PMC11237990; doi:10.1093/jisesa/ieae070)
Supplement: ieae070_suppl_Supplementary_Tables_S1 [file ieae070_suppl_supplementary_tables_s1.pdf]

Supplemental Table S1. Cantaridin levels at different stages in separated rearing adult *Mylabris cichorii*. Adapted from Wang et al. (Wang, 2008) .

Days after emergence	Cantharidin contents (% fresh body weight)	
	Male	Female
0	0.014±0.001	0.097±0.001
5	0.115±0.001	0.114±0.003
10	0.237±0.028	0.119±0.005
15	0.336±0.002	0.089±0.005
20	0.486±0.005	0.058±0.001
25	0.942±0.002	0.048±0.001
30	1.098±0.003	0.064±0.001

Values are means±s.d.
